# Unhealthy Diet Pattern Mediates the Disproportionate Prevalence of Obesity among Adults with Socio-Economic Disadvantage: An Australian Representative Cross-Sectional Study

**DOI:** 10.3390/nu13041363

**Published:** 2021-04-19

**Authors:** Canaan Negash Seifu, Paul Patrick Fahey, Evan Atlantis

**Affiliations:** 1 School of Nursing and Midwifery, Western Sydney University, Penrith, NSW 2751, Australia; e.atlantis@westernsydney.edu.au; 2 School of Health Sciences, Western Sydney University, Penrith, NSW 2751, Australia; p.fahey@westernsydney.edu.au; 3 Translational Health Research Institute, Western Sydney University, Penrith, NSW 2751, Australia; 4 Discipline of Medicine, Nepean Clinical School, Faculty of Medicine and Health, The University of Sydney, Sydney, NSW 2006, Australia

**Keywords:** Mediterranean diet, unhealthy diet, socio-economic disadvantage, obesity

## Abstract

The role of unhealthy dietary pattern in the association between socio-economic factors and obesity is unclear. The aim was to examine the association between socio-economic disadvantage and obesity and to assess mediation effect of unhealthy dietary pattern defined using the Mediterranean diet criteria. The data source was the Australian National Nutrition and Physical Activity Survey. The study sample included 7744 participants aged 18 years and over, 28% of whom had obesity. We used the Australian Socio-Economic Indexes for Areas (SEIFA) classification system for categorizing socio-economic disadvantage; calculated the Mediterranean Diet Score (MDS) using standard criteria; and used measured body mass index to define obesity. We conducted a mediation analysis using log–binomial models to generate the prevalence ratio for obesity and the proportion mediated by the MDS. The most disadvantaged group was associated with higher level of obesity after controlling for covariates (1.40, 95% CI 1.25, 1.56) compared to the least disadvantaged group, and in a dose–response way for each decreasing SEIFA quintile. The relationship between socio-economic disadvantage and obesity was mediated by the MDS (4.0%, 95% CI 1.9, 8.0). Public health interventions should promote healthy dietary patterns, such as the Mediterranean diet, to reduce obesity, especially in communities with high socio-economic disadvantage.

## 1. Introduction

Obesity, defined as a body mass index (BMI) greater than or equal to 30 kg/m^2^, is a rising global health issue and is estimated to have affected 390 million women and 281 million men in 2016 [1]. It is also one of the leading risk factors for an increased fatal and non-fatal disease burden worldwide [2,3]. In high-income countries, obesity is more common in individuals living in areas with high socio-economic disadvantage [4,5]. In Australia, adults in the lowest socio-economic areas were 1.7-fold more likely to have obesity compared to those in the highest socio-economic areas (after adjusting for differences in age structure) for the period 2017 to 2018. Thus, the age-adjusted prevalence of obesity was 37% vs. 26% in men, and 38% vs. 22% in women when comparing the lowest socio-economic areas vs. the highest socio-economic areas [6].

Despite the large-scale investment in community-based projects to prevent weight gain in Australia [7], the prevalence of obesity has increased in recent decades from 19% in 1995 to 31% in 2017–2018, and remains persistently high [8]. There is good evidence that societal changes during this period have likely resulted in population-wide increase in food consumption, especially from energy dense and nutrient poor foods [9,10]. These transitions to obesogenic environments in societies are the aspects which could help explain worldwide changes in BMI and the differences between countries in the current rates of obesity, especially among vulnerable populations.

Unhealthy diet patterns are typically associated with high energy intake specifically from energy dense and nutrient poor foods [11]. A recently published umbrella review showed that higher adherence to the Mediterranean Diet Score (MDS) was consistently associated with a decreased risk of weight gain or obesity in adult populations [12]. Thus, a healthy diet pattern, such as the Mediterranean diet (mainly consisting of fruits, vegetables, cereals and fish, olive oil, and a moderate amount of red wine), may help prevent weight gain [13,14]. While there is good evidence that socio-economic disadvantage significantly predicts both unhealthy dietary pattern and obesity, the association between all three variables is unclear [15,16,17]. To our knowledge, there is very limited evidence on this association [18]. To address this knowledge gap, we aimed to determine whether unhealthy diet pattern, defined using the MDS, mediates the association between socio-economic status and obesity in adults.

## 2. Materials and Methods

We present this study according to the journal’s formatting requirements and the STROBE guidelines for reporting cross-sectional studies [19].

### 2.1. Study Design, Setting, and Participants

We used data from the National Nutrition and Physical Activity Survey (NNPAS)—conducted by the Australian Bureau of Statistics (ABS) in 2011–2012. The survey used a cross-sectional multistage area with an initial sample of 14,400 private dwellings that yielded a final sample of 9435 adults. The methodology has been published in detail elsewhere [20]. For this study, we selected participants who were adults (aged 18 years or over) and not pregnant or breast feeding at the time of the survey (n = 7744).

### 2.2. Ethics

The Australian Health Survey was conducted by the ABS under the Census and Statistics Act 1905 which authorized the ABS to undertake the household interview component of the survey without requiring ethical approval. In October 2011, the Australian Government Department of Health and Ageing’s Departmental Ethics Committee granted ethical approval for the biomedical data collections. Furthermore, written informed consent was obtained from participants for the in-home component and pathology collection center component separately [21]. The de-identified dataset is made available to researchers subject to the requirements of the ABS [22].

### 2.3. Data Sources/Measurement

The NNPAS which is part of the Australian Health Survey (AHS) was conducted from May 2011 to June 2012 in 9500 fully responding private dwellings [20]. Trained ABS interviewers employed a Computer Assisted Personal Interview (CAPI) to collect data from the selected adult member of the household. Twenty-four-hour diet recalls were collected using the five-pass ‘Automated Multiple-Pass Method’ (an automated questionnaire that guides the interviewer through a system designed to maximize respondents’ opportunities for remembering and reporting foods eaten in the previous 24 h). It was developed by the United States Department of Agriculture and it was modified with assistance from Food Standards Australia and New Zealand (FSANZ) to reflect the Australian food supply. Food intake was then coded and classified using “AUSNUT 2011–2013” constructed by FSANZ. For this study, we used dietary data from day one of the 24-h recalls.

Physical measurements were collected towards the end of the NNPAS survey and voluntarily; participants were encouraged to remove shoes and heavy clothing before their measurements were taken. The ABS interviewers used digital scales to measure weight (maximum 150 kg) and recorded it to the nearest 0.1 kg. Height (maximum 210 cm) was measured using a stadiometer and was recorded to the nearest 0.1 cm. Height measurements were repeated on random 10% sample of respondents to validate the measurements.

### 2.4. Variables

All survey questions are listed in the AHS User Guide [23].

### 2.5. Outcome

Obesity and non-obesity were defined by BMI ≥ 30 kg/m^2^ and BMI 18.5–29.9 kg/m^2^ respectively according to the World Health Organization criteria using measured weight and height.

### 2.6. Predictors

Study predictors include Socio-Economic Indexes for Areas (SEIFA) and the MDS. The SEIFA is a measure of socio-economic advantage and disadvantage, created using variables such as income, education, or housing according to residential postcode. The SEIFA was categorized into quintiles. Lower quintiles are indicative of individuals living in areas with higher levels of disadvantage. For the MDS, we followed the method proposed by Tricopoulou et al. [24], since it was one of the most common one reported in the published literature to assess adherence to the Mediterranean diet [12]. A value of 0 or 1 was given to nine components according to sex-specific medians. For potentially healthy components (vegetables, fruits and nuts, and cereal), study participants whose consumption was below the median were assigned a value of 0, and study participants whose consumption was equal to or above the median were assigned a value of 1. For legumes and fish, a value of 1 was given for any intake above zero.

For potentially unhealthy components (meat and dairy products), study participants whose consumption was below the median were assigned a value of 1, and study participants whose consumption was at or above the median were assigned a value of 0. For ethanol, a value of 1 was assigned to men who consumed between 10 and 50 g per day and to women who consumed between 5 and 25 g per day. Lastly, for fat intake, we used the ratio of monounsaturated lipids to saturated lipids and study participants whose consumption was below the median were assigned a value of 0, and study participants whose consumption was at or above the median were assigned a value of 1. The scores for each of the 9 components were summed to give the total MDS which ranged from 0 (minimal adherence to the traditional Mediterranean diet) to 9 (maximal adherence). The NNPAS categorized consumption as discretionary and non-discretionary [20]; in this study, we used food groups from non-discretionary sources to construct the MDS.

### 2.7. Covariates

Our analyses were adjusted for sex (male/female), country of birth (Australia/other English-speaking countries/other), marital status (married or de facto/not married), hours usually worked each week (not in workforce or unemployed/1–24 h/25–39 h/40 h and more), and whether exercise last week met 150 min recommended guidelines (National Physical Activity Guidelines for Australian adults) [20] (met recommended guidelines/did not meet or do not know); smoking status (current smoker/ex-smoker/never smoked), energy density (first tertile/second tertile/third tertile), and self-reported long-term health conditions (no condition/one condition only/multiple conditions) were included. We calculated energy density by dividing the total energy from food by total gram of food (kJ/g).

### 2.8. Bias

The response rate in the NNPAS was 77%. The dietary data in the NNPAS is from a 24-h recall method which can introduce recall bias when study participants forget the food and beverages they have consumed or misreport foods to comply with societal expectations.

### 2.9. Statistical Methods

We present the characteristics of the study participants by obesity and the MDS (Table S1). We tested the difference between the characteristics and the MDS or obesity group using Pearson’s Chi-square tests and their *p*-values. A *p*-value of <0.05 was considered as a statistically significant difference along with clinical importance. Because the prevalence of obesity in the sample was greater than 10% (i.e., 28%), we used log–binary regression models [25] to assess the independent associations with obesity; reported as prevalence ratios with associated 95% confidence intervals.

**Figure 1:**
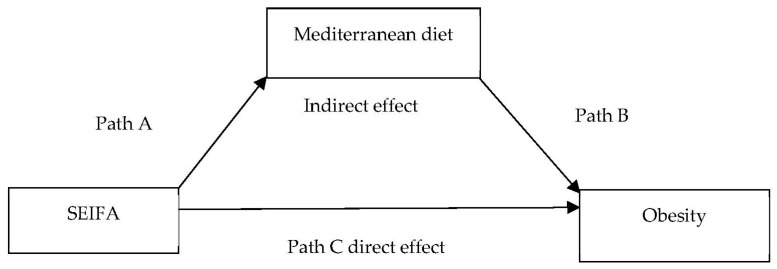
Mediation analysis model of associations between SEIFA and obesity, mediated by adherence to a Mediterranean diet adjusted for covariates. Total effect equals indirect effect + direct effect.

We hypothesized SEIFA would be associated with obesity both directly and indirectly via MDS as a mediator (Figure 1). Therefore, we conducted a mediation analysis to test this hypothesis in a subsample comparing lowest vs. highest SEIFA. We performed the analysis by fitting a log–binomial model for the outcome (obesity, yes = 1 and no = 0) and the MDS-mediator (0–4 = 1 and 5–9 = 0). We obtained prevalence ratios of direct effect, indirect (mediated) effect, and total effect. We calculated percentages by multiplying the estimate with 100%. We conducted the statistical analyses using IBM SPSS Statistics 25 (Statistical Package for the Social Sciences) and the ‘mediation’ package [26] in R 4.0.3 to perform the mediation analysis.

## 3. Results

The study sample included 7744 adults. Of these, 2185 (28.2%) were classified with obesity. From these adults, 1549 (70.9%) had lower MDS (0–4), 481 (22.0%) lived in most disadvantaged areas (SEIFA-lowest 20%), 1217 (55.7%) were married, and 834 (38.2%) were not in workforce or unemployed (Table 1).

A total of 5084 (65.7%) participants had lower MDS (0–4); of these, 1018 (20.0%) participants lived in most disadvantaged areas and 1822 (35.8%) were not in workforce or unemployed (Table S1).

In terms of the association between SEIFA and obesity, the prevalence of obesity increased with socio-economic disadvantage categories in a linear way, independent of covariates (Models 1–6) (Table 2). The strength of this relationship slightly weakened, but remained robust even after adjustment for all covariates (Model 6). Furthermore, SEIFA was associated with the MDS (Table S2).

To do the mediation analysis (Table 3), we first assessed the association between SEIFA and obesity (direct effect); lowest SEIFA (most disadvantaged) was associated with obesity. Second, we examined path A and path B; the lowest SEIFA (most disadvantaged) was associated with lower MDS (0–4) (Path A) and lower MDS (0–4) was associated with higher prevalence of obesity (Path B), respectively. The results showed a significant effect (PR 1.13, 95% CI 1.08, 1.19) of lowest SEIFA associated with lower MDS (0–4) (Path A), and a significant effect (PR 1.19, 95% CI 1.10, 1.29) of lower MDS (0–4) associated with obesity (Path B). Finally, SEIFA had an indirect effect (PR 1.00, 95% CI 1.00, 1.01) on obesity and 4.0% of the total effect of lowest SEIFA (most disadvantaged) on obesity is due to SEIFA’s impact on the MDS.

## 4. Discussion

### 4.1. Main Finding

To our knowledge, this is the first study to examine the mediating role of unhealthy diet pattern, defined using low MDS, in the association between socio-economic disadvantage and obesity. First, we found that socio-economic disadvantage was independently associated with increased prevalence ratios for obesity that ranged from 18% to 40% percentage points with each decreasing SEIFA quintile group in a dose–response way (Model 6. Table 2). This is consistent with several studies from high income countries like the USA, the UK, and Canada [27,28,29]. Second, we found that socio-economic disadvantage was independently associated with low MDS (Model 5, in Table S2). Third, our mediation model showed that at least 4% of the association between the lowest SEIFA (highest disadvantage) and obesity prevalence was mediated by low MDS (i.e., fewer elements of the MDS). These findings suggest that a significant proportion of the prevalence of obesity in the community could have been theoretically prevented with effective public health policy and/or community-based interventions targeting unhealthy dietary patterns. For instance, interventions focusing on a ‘healthy dietary pattern’ like the MDS could have theoretically prevented more than 200,000 cases of obesity, which would have yielded substantial health and economic benefits for Australians [30,31].

Our finding demonstrating a mediation role of unhealthy diet confirms, for the first time, a plausible mechanism explaining the increased risk of obesity associated with socio-economic disadvantage. There is good evidence showing that exposure to socio-economic disadvantage is associated with an increased consumption of nutrient-poor and energy-dense foods (Path A); i.e., consuming more processed meat, refined grain, and sweets, but less fruit and vegetables, whole grains, or fish [15,32,33]. This phenomenon is likely explained by the high cost and low availability barriers to accessing healthy foods in societies around the world [34,35]. This theory is supported by findings from a cohort study which found that the higher daily cost of following a Mediterranean dietary pattern compared with a western dietary pattern was a predictor of weight gain among Spanish university graduates [36]. Furthermore, a systematic review suggests that environmental factors such as accessibility to supermarkets/takeaway outlets or residing in a socioeconomically disadvantaged area may contribute to obesogenic dietary behavior [37]. Finally, there is evidence suggesting that when food retail environments are unstable, low income areas are more affected by obesity than high income areas [38]. Thus, interventions on taxation of unhealthy foods supplemented with other policies such as subsidizing healthy foods could be helpful in preventing obesity [39,40,41]. Strategies focusing on fiscal measures (e.g., food vouchers for disadvantaged communities) and dietary standards [42] and healthy food promotion in retail environments may encourage healthy eating behavior in communities [43].

There is also good evidence showing an association between MDS and obesity (Path B). Unhealthy diet pattern has been associated with an increased risk of weight gain [44] or obesity [45,46]. By contrast, our recently published umbrella review of 16 systematic reviews showed that high adherence to the MDS was consistently associated with a decreased risk of obesity [12]. Previous research has shown that the nutrient-rich low-energy composition of the diet pattern defined using a high MDS [47] was associated with preventing weight gain in the long term [48]. Collectively, this evidence suggests that it may help slow or even reverse the rising prevalence of obesity in some countries. In addition, clinical trials show that there are health benefits of consuming a Mediterranean diet pattern beyond body weight status, such as better cardiovascular health [49,50] and overall mortality [51].

### 4.2. Study Strength and Limitation

The main strength of our study is that we used a subsample from a nationally conducted survey which collected measured height and weight data for accurate classification of obesity status. However, several limitations are noteworthy. First, we used dietary data from day one of 24-h recalls, which may not reflect the usual intake of study participants. Second, because of the cross-sectional design of the study, establishing temporality in exposure, mediator, and outcome variables means that interpretations of our findings should be cautious. Finally, there is no universal agreement on how to measure or define the Mediterranean diet.

## 5. Conclusions

Our findings suggest that unhealthy diet patterns could partially mediate the association between socio-economic disadvantage and obesity. Public health policy and interventions to increase population-wide consumption of healthy diet patterns, such as the Mediterranean, in communities, especially those most disadvantaged groups in the society could help reduce the socio-economic inequalities of obesity.

## Figures and Tables

**Table 1:** Participant characteristics by obesity category in the NNPAS 2011 to 2012 (n = 7744).

		Obesity Status		*p*-Value
		Number (Percent)		
		Without Obesity BMI 18.5–29.9 kg/m^2^ n = 5559	With Obesity BMI ≥ 30 kg/m^2^ n = 2185	
MDS ^1^	0–4	3535 (63.6)	1549 (70.9)	<0.001
	5–9	2024 (36.4)	636 (29.1)	
SEIFA ^2^	Most disadvantaged	970 (17.4)	481 (22.0)	<0.001
	Second quintile	1057 (19.0)	523 (23.9)	
	Third quintile	1134 (20.4)	418 (19.1)	
	Fourth quintile	1001 (18.0)	370 (16.9)	
	Least disadvantaged	1397 (25.1)	393 (18.0)	
Sex	Male	2718 (48.9)	1030 (47.1)	0.1648
	Female	2841 (51.1)	1155 (52.9)	
Country of birth	Australia	3849 (69.2)	1634 (74.8)	<0.001
	Other English-speaking countries	700 (12.6)	269 (12.3)	
	Other countries	1010 (18.2)	282 (12.9)	
Marital status	Married/de facto	2899 (52.1)	1217 (55.7)	0.005
	Not married	2660 (47.9)	968 (44.3)	
Hours usually worked each week	Not in workforce/unemployed	1834 (33.0)	834 (38.2)	<0.001
	1–24 h	752 (13.5)	247 (11.3)	
	25–39 h	1169 (21.0)	434 (19.9)	
	40 h and more	1804 (32.5)	670 (30.7)	
Energy density ^3^	First tertile	1764 (31.7)	817 (37.4)	<0.001
	Second tertile	1885 (33.9)	697 (31.9)	
	Third tertile	1910 (34.4)	671 (30.7)	
Smoking status	Current smoker	1084 (19.5)	391 (17.9)	<0.001
	Ex-smoker	1700 (30.6)	844 (38.6)	
	Never smoked	2775 (49.9)	950 (43.5)	
Whether exercise last week, met 150 min recommended guidelines	Met recommended guidelines	3002 (54.0)	935 (42.8)	<0.001
	Did not meet or do not know	2557 (46.0)	1250 (57.2)	
Long-term conditions	No condition	4413 (79.4)	1377 (63.0)	<0.001
	One condition only	773 (13.9)	473 (21.6)	
	Multiple conditions	373 (6.7)	335 (15.3)	

^1^ Mediterranean diet score, ^2^ Index of Relative Socio-Economic Disadvantage—2011—SA1—Quintiles—National, ^3^ Energy density- First tertile (0.00–2.21); Second tertile (2.22–3.08); Third tertile (3.09–14.09).

**Table 2:** Multivariable adjusted association between SEIFA and obesity in the NNPAS 2011 to 2012 (n = 7744).

SEIFA ^1^	Model 1	Model 2	Model 3	Model 4	Model 5	Model 6
	PR (95%CI)	PR (95%CI)	PR (95%CI)	PR (95%CI)	PR (95%CI)	PR (95%CI)
Most disadvantaged	1.51(1.35, 1.69) ***	1.51(1.35, 1.69) ***	1.48(1.32, 1.66) ***	1.46(1.30, 1.63) ***	1.46(1.30, 1.63) ***	1.40(1.25, 1.56) ***
Second quintile	1.51(1.35, 1.69) ***	1.51(1.35, 1.68) ***	1.45(1.30, 1.62) ***	1.43(1.28, 1.60) ***	1.43(1.28, 1.59) ***	1.38(1.24, 1.54) ***
Third quintile	1.23(1.09, 1.38) **	1.22(1.09, 1.38) **	1.20(1.06, 1.35) **	1.19(1.05, 1.34) **	1.18(1.05, 1.33) **	1.18(1.05, 1.32) **
Fourth quintile	1.23(1.09, 1.39) **	1.24(1.09, 1.40) **	1.23(1.09, 1.38) **	1.22(1.08, 1.38) **	1.22(1.08, 1.37) **	1.20(1.06, 1.35) **
Least disadvantaged	Reference	Reference	Reference	Reference	Reference	Reference

Notes: Model 1, unadjusted; Model 2, adjusted for sex, country of birth, marital status, hours usually worked each week; Model 3, adjusted for whether exercise last week met 150 min recommended guidelines, smoking status; Model 4, adjusted for energy density; Model 5, adjusted for MDS; Model 6, adjusted for long-term conditions. ^1^ Index of Relative Socio-Economic Disadvantage—2011—SA1—Quintiles—National. ** *p* < 0.01, *** *p* < 0.001.

**Table 3:** Mediation analysis of the association between SEIFA and obesity mediated by adherence to Mediterranean diet (n = 7744).

	PR (95%CI)	Proportion Mediated by MDS % (95% CI)
Path A (SEIFA→MDS)	1.13(1.08, 1.19) ***	
Path B (MDS→Obesity)	1.19(1.10, 1.29) ***	
Path C direct effect (SEIFA→Obesity)	1.09(1.06, 1.12) ***	
Indirect effect (SEIFA→Obesity mediated by MDS)	1.00(1.00, 1.01) ***	
Total effect (SEIFA→MDS→Obesity)	1.09(1.06, 1.13) ***	4.0(1.9, 8.0)

Notes: SEIFA—(5 categories), reference—Least disadvantaged; MDS- (0–4) vs. (5–9); Obesity- Yes = 1 vs. No = 0. All models were adjusted for sex, country of birth, marital status, hours usually worked each week, whether exercise last week met 150 min recommended guidelines, smoking status, energy density and long-term conditions. *** *p* < 0.001.

## Data Availability

Publicly available datasets were analyzed in this study. These data can be found here, subject to the ABS requirements https://www.abs.gov.au/websitedbs/D3310114.nsf/home/MicrodataDownload, accessed on 23 August 2019.

## Supplementary Materials

The following are available online at https://www.mdpi.com/article/10.3390/nu13041363/s1, Table S1: Participant characteristics by the MDS in the NNPAS 2011 to 2012 (n = 7744); Table S2: Multivariable adjusted association between SEIFA and the MDS in the NNPAS 2011 to 2012 (n = 7744).
