# Supplementary material for: Unhealthy Diet Pattern Mediates the Disproportionate Prevalence of Obesity among Adults with Socio-Economic Disadvantage: An Australian Representative Cross-Sectional Study
Source: Nutrients. 2021 Apr 19;13(4):1363. doi: 10.3390/nu13041363 (PMC8072565; doi:10.3390/nu13041363)
Supplement: Supplementary file 1 [file nutrients-13-01363-s001.zip › nutrients-1142374-supplementary.pdf]

Supplementary Materials

Table S1. Participant characteristics by the MDS in the NNPAS 2011 to 2012 (*n* = 7744).

		**MDS Categories Number (Percent)**		
		**0–4 (fewer elements of Mediterranean diet) *n* = 5,084**	**5–9 (more elements of Mediterranean diet) *n* = 2,660**	***p*-value**
SEIFA	Most disadvantaged	1,018 (20.0)	433 (16.3)	<0.001
	Second quintile	1,101 (21.7)	479 (18.0)	
	Third quintile	1,038 (20.4)	514 (19.3)	
	Fourth quintile	866 (17.0)	505 (19.0)	
	Least disadvantaged	1,061 (20.9)	729 (27.4)	
Sex	Male	2,376 (46.7)	1,372 (51.6)	<0.001
	Female	2,708 (53.3)	1,288 (48.4)	
Country of birth	Australia	3,768 (74.1)	1,715 (64.5)	<0.001
	Other English speaking	642 (12.6)	327 (12.3)	
	Other	674 (13.3)	618 (23.2)	
Marital status	Married/ de facto	2,659 (52.3)	1,457 (54.8)	0.039
	Not married	2,425 (47.7)	1,203 (45.2)	
Hours usually worked each week	Not in workforce/unemployed	1,822 (35.8)	846 (31.8)	0.003
	1-24 hrs	657 (12.9)	342 (12.9)	
	25-39 hrs	1,033 (20.3)	570 (21.4)	
	40 hrs and more	1,572 (30.9)	902 (33.9)	
Energy density[3]	First tertile	1,727 (34.0)	854 (32.1)	<0.001
	Second tertile	1,618 (31.8)	964 (36.2)	
	Third tertile	1,739 (34.2)	842 (31.7)	
Smoking status	Current smoker	1,096 (21.6)	379 (14.2)	<0.001
	Ex-smoker	1,612 (31.7)	932 (35.0)	
	Never smoked	2,376 (46.7)	1,349 (50.7)	
Exercise	Met recommended guidelines	2,436 (47.9)	1,501 (56.4)	<0.001
	Did not meet or don't know	2,648 (52.1)	1,159 (43.6)	
Obesity category	No	3,535 (69.5)	2,024 (76.1)	<0.001
	Yes	1,549 (30.5)	636 (23.9)	
Long-term health conditions	No condition	3,799 (74.7)	1,991 (74.8)	0.992
	One condition only	819 (16.1)	427 (16.1)	
	Multiple conditions	466 (9.2)	242 (9.1)	

[1] Mediterranean diet score. [2]Index of Relative Socio-Economic Disadvantage—2011—SA1—Quintiles—National. [3]Energy density- First tertile (0.00–2.21); Second tertile (2.22–3.08); Third tertile (3.09–14.09).

Table S2. Multivariable adjusted association between SEIFA and the MDS in the NNPAS 2011 to 2012 (n = 7744).

SEIFA [1]	Model 1<br>PR (95%CI)	Model 2<br>PR (95%CI)	Model 3<br>PR (95%CI)	Model 4<br>PR (95%CI)	Model 5<br>PR (95%CI)
Most disadvantaged	1.18(1.12, 1.25) ***	1.18(1.12, 1.24) ***	1.13(1.07, 1.19) ***	1.13(1.07, 1.18) ***	1.13(1.07, 1.19) ***
Second quintile	1.18(1.12, 1.24) ***	1.16(1.11, 1.22) ***	1.13(1.08, 1.19) ***	1.13(1.08, 1.19) ***	1.14(1.08, 1.19) ***
Third quintile	1.13(1.07, 1.19) ***	1.12(1.06, 1.18) ***	1.09(1.04, 1.15) ***	1.09(1.04, 1.15) **	1.09(1.04,1.15) ***
Fourth quintile	1.07(1.01, 1.13) *	1.07(1.01, 1.13) *	1.05(1.00, 1.11)	1.05(1.00, 1.11)	1.05(1.00, 1.11)
Least disadvantaged	Reference	Reference	Reference	Reference	Reference

Notes: Model 1, unadjusted; Model 2, adjusted for sex, country of birth, marital status, hours usually worked each week; Model 3, adjusted for whether exercise last week met 150 minutes recommended guidelines, smoking status; Model 4, adjusted for energy density; Model 5, adjusted for long-term conditions. [1]Index of Relative Socio-Economic Disadvantage—2011—SA1—Quintiles—National. * $p < 0.05$ ** $p < 0.01$ *** $p < 0.001$.
